# Competing challenges for immigrant seniors: Social isolation and the pandemic

**DOI:** 10.1177/08404704211009233

**Published:** 2021-05-13

**Authors:** Shanthi Johnson, Juanita Bacsu, Tom McIntosh, Bonnie Jeffery, Nuelle Novik

**Affiliations:** 1 School of Public Health, 3158 University of Alberta, Edmonton, Alberta, Canada.; 2 Department of Psychology, SPHERU, University of Saskatchewan, Saskatoon, Saskatchewan, Canada.; 3 Department of Politics and International Studies, SPHERU, 6846 University of Regina, Regina, Saskatchewan, Canada.; 4 Faculty of Social Work, SPHERU, 6846 University of Regina, Regina, Saskatchewan, Canada.

## Abstract

The pandemic has exposed and amplified complex and complicated health and societal challenges while offering immense opportunities to transform societies to improve health for all. Social isolation is a challenging and persistent issue experienced by many older adults, especially among immigrant and refugee seniors. Unique risk factors such as racism, discrimination, language barriers, weak social networks, and separation from friends and family predispose immigrant and refugee seniors to a higher risk of social isolation. The pandemic has magnified the unique risks and has highlighted the differential health and economic impacts. This article examines social isolation among immigrant and refugee seniors in Canada by focusing on the policy context, available programs and services to reduce social isolation, and the conceptualization and measurement considerations for advancing research to address social isolation among this growing population. Drawing on specific examples, we discuss immigration, aging, and social isolation within the context of Canada. While our article focuses on Canada as a case study, our discussion has relevancy and implications for other high-income countries with aging immigrant and refugee populations. In moving forward, we argue that a more complete and targeted understanding of social isolation is essential to informing program and policy development to support immigrant and refugee seniors in Canada and beyond. The transformation needed in our societies to create health for all requires strong equity and determinants of health perspective and a systems approach beyond health to ensure lasting change.

## Introduction

The pandemic has impacted all segments of the population and is particularly detrimental to those with differential risks and impacts—such as seniors, women and racialized individuals/minority populations, those with disabilities, those who live in crowded housing, or those suffering from addictions and other mental health issues. A recent study by Statistics Canada reported that immigrants and refugees are more likely to be worried about the economic and social impacts of the pandemic than the general population.^1^ Recent data also show that older adults aged 65 and older, people with chronic conditions and/or cognitive impairment, and minority populations are disproportionately affected by the pandemic.^2,3^ Immigrant and refugee seniors are especially vulnerable to the social, physical, and psychological issues from the pandemic as they face a heightened risk of morbidity and mortality from the pandemic.^4^ A resilient and healthy future depends on our processes/programs/policies strengthened by our collective experiences emerging from the pandemic and other macro societal health challenges. The World Health Organization has emphasized that speed, scale, and equity must be our guiding principles as we forge ahead to create transformative solutions.^5^

Social isolation is a challenging and persistent global issue experienced by many older adults, especially among immigrant and refugee seniors.^6,7^ While social isolation is common among older adults, further amplified through the effects of the pandemic, unique risk factors such as racism, discrimination, language barriers, weak social networks, and separation from friends and family predispose immigrant and refugee seniors to a higher vulnerability for social isolation.^5,8,9^ Social isolation is commonly defined as the level and frequency of one’s interactions with others.^10^ Loneliness is different than social isolation as it reflects the subjective feeling that results from inadequate social connections and feeling disconnected from others.^6^

In order to effectively address social isolation among this vulnerable population, understanding Canada’s immigration system and policies is critical. Existing programs and services to reduce social isolation provide valuable insight into organizational capacity, innovation, and community resources while identifying significant gaps. They also highlight regional disparity and the need for more evidence-informed initiatives. Although social isolation among immigrant and refugee seniors is a global issue, there is limited discourse on preventative strategies and action-based research to support this growing population.^5,9^ There are key challenges related to the conceptualization and measurement of social isolation. While social isolation and loneliness are inherently different, they are often studied interchangeably.^11^ It is also important to recognize that immigrant and refugee seniors are themselves a heterogenous group. Policy frameworks require an understanding of the need to accommodate those differences and the extent to which they put people at risk of social isolation.

This article examines social isolation among immigrant and refugee seniors in Canada by focusing on the policy context, programs and services for reducing social isolation, and issues of conceptualization and measurement for advancing research to address social isolation among this growing population. Drawing on specific examples, we discuss immigration, aging, and social isolation within the context of Canada. While our article focuses on Canada and immigrant and refugee seniors specifically, some of the discussion has relevancy and implications for older adults in general and other countries.

## Policy context: Immigration in Canada

Canada is a multicultural society with an increasing number of immigrant and refugee seniors. In 2011, 21% of the total Canadian population was foreign-born with a projected increase to 40% in 2055. Among seniors, 30% were foreign-born, compared to 21% of the total population.^8^

Immigration policy in Canada is one of a small number of enumerated constitutional powers that are shared between the national and provincial governments. Immigration is a shared jurisdiction with federal paramountcy. Any provincial or territorial law in the area of immigration must be consistent with federal law, and any disputes between federal and provincial legislation would be justiciable in the courts. The primary federal legislation is the *Immigration and Refugee Protection Act*, 2001, along with the *Citizenship Act* and relevant provisions of the *Criminal Code of Canada*. The bulk of federal immigration policy is the purview of the current Ministry of Immigration, Refugees, and Citizenship Canada. Responsibility for immigration and refugee policy in the provinces is allocated to different ministries which occasionally meet as the Federal/Provincial/Territorial Forum for Ministers Responsible for Immigration.

Typically, most immigrant and refugee seniors have lived in Canada for a long time, since people often migrate when they are relatively young.^7^ The majority of immigrant seniors in Canada live in Ontario, British Columbia, and Québec.^12^ Provinces and regions may introduce special incentives and programs designed to attract immigrants to specific parts of the country; however, these incentives must not interfere with federal legislation on immigration law.^13^

Canada has been quite successful in integrating large numbers of immigrants and refugees. Second- or third-generation Canadians showed few significant differences in terms of socio-economic status or educational attainment from those with deeper roots in the country.^13,14^

In comparison to Canadian-born seniors, new immigrant and refugee seniors face a higher risk of social isolation.^9^ An important risk factor for social isolation among this population may be related to the specific category under which they arrive. Immigrants may find themselves with fewer supports because they are presumed to have the capacity to manage their own integration. Refugees sponsored by the government are often assisted by government social workers with large, diverse caseloads and can struggle to get needs met in a timely manner before the sponsorship ends. Privately sponsored refugees (note 1) may have more “personal” support, but it also comes from people and organizations with a huge variation in skill sets for the complicated task of accessing the best services to meet refugees’ diverse needs.

Social isolation among immigrant and refugee seniors can arise in a number of circumstances and from a variety of causes. What is common among those different manifestations of social isolation is that it often arises unseen by government agencies, immigrant communities, and even families. Identifying socially isolated older adults is one of the biggest challenges.^7^ Immigrants and refugees are also diverse with different countries of origin, cultures, familial customs, living arrangements, and languages. As the population ages and the proportion of immigrant and refugees increase, federal and provincial policy-makers in Canada are faced with new challenges to adequately address issues of social isolation.

## Programs and services

In Canada, various programs and services exist to reduce social isolation among immigrant and refugee seniors. In a recent environmental scan, 40 programs and services to support this population across the country were identified.^7,15^ The development of programs and services to support socially isolated immigrant and refugee seniors is promising; however, the geographic distribution and number of programs compared to need were limited as shown in the table (Table 1).

**Table 1.** Geographic distribution of programs and services

Province/Territory	Program/Service
Alberta	8
British Columbia	12
Manitoba	1
New Brunswick	–
Newfoundland	1
Northwest Territories	–
Nova Scotia	1
Nunavut	–
Prince Edward Island	–
Ontario	14
Quebec	2
Saskatchewan	1
Yukon	–

Service and program gaps were noted for immigrant and refugee seniors living outside of large urban centres as the majority were offered in large, urban centres.^16,17^ No specific programs or services were identified in rural or remote communities, suggesting that newcomer seniors would be relatively disadvantaged in these areas.^11^ Further research is required to examine social isolation and immigrant and refugee seniors living in rural and remote communities.

Programs and services for immigrant and refugee seniors often focused on supports for English Language training, settlement services, social support, computer classes, and information to access available resources. These programs and services typically incorporated opportunities to meet people and build friendships to help prevent and reduce social isolation.^18^

The evidence suggests a need for more programs and services to target vulnerable subgroups among immigrant and refugee seniors. Vulnerable subgroups identified with service gaps were immigrant and refugee senior caregivers^11^ and those with health conditions, disabilities, and/or with hearing or visual impairments.

Organizations often provided activities with similar goals and objectives related to social inclusion.^19,20^ However, only a few of the providers appeared to be working in partnership with other organizations to support immigrant and refugee seniors. Programs were typically supported by limited grants and government funding.^8,11,15^ Increased collaboration between the service providers could strengthen coordination for capacity-building and sustainability to support programs and services over time.

Individuals and groups working to reduce social isolation among immigrant and refugee seniors require access to an integrated, up-to-date guide of existing programs and services available. In moving forward, we recommend that an on-line resource featuring a nation-wide perspective be developed and maintained with current information on available programs and services to support simplified access of information for supporting seniors. While the programs and services discussed here were specific to immigrant and refugee seniors, the issue of social isolation impacts the lives of many seniors. The shared and unique risk factors should be considered in developing, implementing, and evaluating the outcomes and impacts of social isolation programs.

## Conceptualization and measurement challenges

The conceptualization of social isolation of seniors varies considerably across different disciplines with no clear consensus on either the definition or the best way to measure social isolation.^21,22^ Some researchers consider that indicators of social isolation require more objectivity including dimensions such as the number and frequency of social contacts or meaningful ties and fulfilling relationships.^23–25^ This is interconnected with indicators of loneliness which are typically more subjective including, for example, an assessment of people’s feelings and cognitions surrounding the quantity and quality of social contacts and relationships.^10^ Others suggest that indicators and measures of social isolation should be both objective and subjective using, for example, measures such as lack of social ties or networks along with an assessment of the perceived level of isolation that arises from this objective state.^26^

There is a predominant focus on measuring loneliness rather than focusing specifically on social isolation. There are two frequently used loneliness scales that have been proven to be reliable and valid^27^: the revised UCLA Loneliness Scale^28^ and the de Jong Gierveld Loneliness Scale.^23^ The de Jong Gierveld scale is the more commonly used measure of loneliness.^27,29^ The six-item version of this measure has been used in the exploration of older adult immigrant populations specifically^30,31^ and has been paired with the abbreviated 6-item Lubben Social Network Scale,^32^ which is one of the most frequently used validated measures of social network in studies examining social isolation among seniors.^22^ This is a promising measurement combination where both loneliness and social networks are recognized as important elements in examining social isolation. Social isolation and loneliness are however different and more research is needed to focus specifically on understanding social isolation among seniors in general and immigrant and refugee seniors in particular.

## Moving forward to reduce social isolation in Canada and beyond

Social isolation is complex, multidimensional, and contextual.^33^ Both quantitative and qualitative aspects of social isolation are necessary to capturing a more nuanced picture of social isolation among seniors.^34^ While social isolation may be influenced by individual-level factors, it is also influenced by structural barriers that can affect subgroups of seniors in different ways.^7^ As such, social isolation measures that incorporate the experience of seniors who are immigrants or refugees require particular attention.^11^ There are specific factors that warrant further investigation in order to reduce social isolation among immigrant and refugee seniors:

### English and French proficiency

A large number of immigrant and refugee seniors have limited English or French language proficiency^9^ which can affect both their ability to participate in social interactions outside the family and home and their social relationships with family members.^35,36^ Some may have difficulty communicating with their grandchildren due to a language barrier^37^ or they may become overly reliant on their grandchildren for translation services. As noted by Maiter,^38^ the lack of comfort with the language can affect the ability to read newspapers or use public transportation, both of which can be barriers to participating more fully in social activities.

### Cultural differences

Cross-cultural variation should be also considered in developing measures of social isolation.^21,39^ Biordi and Nicholson^40^ suggest that a cultural screening should be built into social isolation measures in order to recognize the differences among cultures in terms of how social relations are organized. Hossen^37^ suggests that in some cultures, collectivism or family orientation is more highly valued than individualism and that this may not necessarily fit with the prevailing values in the host country.

### Duration of residence in host country

Alegria et al.^41^ suggest that in the process of immigration, 10 years is an indicator of adaptation. It will be helpful to determine whether seniors at an early stage of immigration are more at risk of social isolation than those who have been in the host country for a longer period of time.^42^

### Status in host country

The status of the older adult in the host country may be an important indicator of social isolation.^37^ Refugee claimants may face a higher risk of being socially isolated, given that they have fewer family members or other community members when relocating to the host country.^43^

### Stigma/discrimination

Discrimination on the basis of race or ethnicity may lead to social isolation,^5,44^ where rather than exposing themselves to judgmental remarks, immigrant or refugee seniors may isolate themselves from situations where they will experience discrimination.^45^

### Loss of social roles

Loss of social roles could prove to be a useful analytical factor in examining social isolation for this group. Migration affects the accustomed family patterns which lead to changes in family members’ roles and responsibilities. Consequently, immigrant and refugee seniors may lose their major roles in the family which they used to play, which may make them vulnerable to isolation.^37^

### Financial status

A large proportion of immigrant and refugee seniors experience difficulty in finding employment due to the interplay of various social determinants of health, including limited language proficiency and health and mobility challenges.^10,34^ They may become financially dependent on their children or grandchildren, which can pose limitations on their ability to participate in activities outside of the home.^20,46,47^

### Living arrangements

A large number of immigrant seniors live with their children when moving to the host country.^19^ This can be a positive influence on reducing feelings of social isolation but intergenerational conflict and change in the older adult’s social role can increase feelings of social isolation.^37,48^

### Pandemic and other emergencies

The pandemic has further exacerbated social isolation among older adults due to their higher susceptibility to the disease and the public health measures requiring shelter in place, gathering restrictions, and physical distancing. Policies and programs adopted for the pandemic need to take into account the fact that the pandemic hits certain populations inequitably, such as immigrant and refugee seniors, given the unique risk factors.

## Conclusion

Canada’s population is aging, and the immigrant and refugee are increasing at a proportionally higher rate. In this context and with the added challenge of the pandemic, social isolation must occupy a significant place in the policy dialogue on seniors and healthy aging. While social isolation is experienced by many seniors, further amplified through the pandemic, immigrant and refugee seniors often present with a host of unique risk factors that must be considered including language proficiency, experiences of racism and discrimination, separation from their family, fewer cultural and other social connections, and financial dependence on their children. All of this is compounded depending on when an individual became an immigrant or refugee as compared with those who came much later in life being more vulnerable to social isolation and less able or likely to have access to necessary supports.

The complexity of the issue requires a broad lens from the perspective of the policy context, available programs and services for addressing social isolation, and research including conceptualization and measurement considerations. Current programs and services available to prevent and reduce social isolation do provide insight and innovative approaches—but also highlight significant gaps that raise questions about equity and universality. There has been a paucity of research focusing on immigration, aging, and social isolation in Canada. Reducing social isolation requires partnership and collaboration with immigrant and refugee seniors, and the development of evidence-informed research to address key factors such as culture and language proficiency.

Whereas there is a lack of research specifically focused on the pandemic impacts on social isolation among immigrant and refugee seniors, there are many directions in the programs and policy context that can be inferred. Within the policy context, it would be useful to identify high-risk seniors, collaborate with agencies and families, and spread accurate and timely information. During the pandemic, it is imperative to adapt interventions and make them accessible in a way that protects seniors. Specific efforts need to be made to strengthen connections between service agencies and the seniors at risk of social isolation using remote and digital modalities. The digital divide and/or lack of internet access or electronic devices may be a problem for seniors and should be considered for alternate modalities of support. Innovations to meet the health and social needs of the growing population of older adults, particularly those for immigrant and refugee seniors require fiscal and policy commitments to expanded access and distribution of devices and the means to instruct seniors in how to use laptops, tablets, phones, and apps.

In moving forward, a more complete understanding of social isolation is essential to informing policy, programs, and research development to support diverse older adults in Canada and beyond. In addition, evidence-informed action should be pursued with speed, scale, and equity to address this health and societal challenge.

## Note

1. Canada has a bifurcated refugee settlement process. The federal government sets numeric limits on the numbers of refugees it will settle at public cost each year (which can be adjusted to meet specific refugee crises that arise internationally). At the same time, it allows private individuals and not-for-profit organizations (eg, charities, churches, community-based organizations) to sponsor refugees in a process where the sponsor assumes the financial and other burden associated with settlement and integration of those they sponsor.
